# Mechanisms of chemotherapy resistance in ovarian cancer

**DOI:** 10.20517/cdr.2021.147

**Published:** 2022-04-03

**Authors:** Mylena Ortiz, Emma Wabel, Kerry Mitchell, Sachi Horibata

**Affiliations:** ^1^Precision Health Program, Michigan State University,766 Service Road, East Lansing, MI 48824, USA.; ^2^Department of Pharmacology and Toxicology, College of Human Medicine, Michigan State University, East Lansing, MI 48824, USA.; ^#^Authors contributed equally.

**Keywords:** Drug resistance, ovarian cancer, cisplatin, carboplatin, paclitaxel, olaparib, niraparib, bevacizumab

## Abstract

Ovarian cancer is one of the most lethal gynecologic cancers. The standard therapy for ovarian cancer has been the same for the past two decades, a combination treatment of platinum with paclitaxel. Recently, the FDA approved three new therapeutic drugs, two poly (ADP-ribose) polymerase inhibitors (olaparib and niraparib) and one vascular endothelial growth factor inhibitor (bevacizumab) as maintenance therapies for ovarian cancer. In this review, we summarize the resistance mechanisms for conventional platinum-based chemotherapy and for the newly FDA-approved drugs.

## INTRODUCTION

Ovarian cancer is one of the most lethal gynecologic cancers, with projected 21,410 cases and 13,770 deaths in the United States in 2021^[1]^. The standard treatment for ovarian cancer is platinum-based chemotherapy (carboplatin or cisplatin) in combination with paclitaxel, and it has remained the same for the past two decades. Most patients are initially responsive to these treatments; however, relapse occurs in around 80% of women due to platinum resistance, causing the need to comprehend its molecular mechanisms to improve treatment efficacy and patient survival^[2]^.

Cisplatin was first synthesized in 1844 by Michele Peyrone, but it was not until 1965 that Barnett Rosenberg from Michigan State University discovered that cisplatin inhibits cell division. Since then, cisplatin has been widely used for the treatment of bladder, lung, head and neck, testicular, and ovarian cancers^[3]^. In 1978, it became the first FDA-approved platinum-based compound for cancer treatment^[4]^. Subsequently, a second-generation platinum-drug, carboplatin, was developed and became FDA-approved in 1989^[5,6]^. 

In the 1960s, the National Cancer Institute and the U.S. Department of Agriculture collaborated to identify potential cytotoxic anticancer properties from 115,000 plant extracts^[7]^. From screening these samples, Arthur Barclay identified cytotoxic properties in bark extract from the Pacific yew tree, *Taxus brevifolia*,in 1964. Three years later, the active ingredients from *Taxus brevifolia* were identified and named as taxol. A fully synthetic version of the drug, called paclitaxel, was FDA-approved for use as a chemotherapeutic agent for ovarian cancer in 1992. Clinical trials in the early 2000s have shown improved outcomes in women with relapsed ovarian cancer when treated with paclitaxel in addition to platinum-based chemotherapy^[8]^. Since then, the standard treatment of ovarian cancer patients continues to be platinum-based chemotherapy in combination with paclitaxel.

Recently, there have been new advancements in treatment recommendations for ovarian cancer patients with the emergence of three new FDA-approved chemotherapeutic drugs. In 2018, the first poly (ADP-ribose) polymerase inhibitor (PARPi), olaparib, was FDA-approved as maintenance therapy for ovarian cancer, followed by the approval of niraparib (another PARPi) in 2020. In 2020, a vascular endothelial growth factor inhibitor (VEGFi), bevacizumab, was also FDA-approved as another maintenance therapy for ovarian cancer. In this review, we will discuss the resistance mechanisms of conventional chemotherapies and provide insights into the resistance mechanisms against the recently FDA-approved chemotherapeutic drugs for ovarian cancer.

## MECHANISMS OF RESISTANCE TO PLATINUM AGENTS

**Figure 1:**
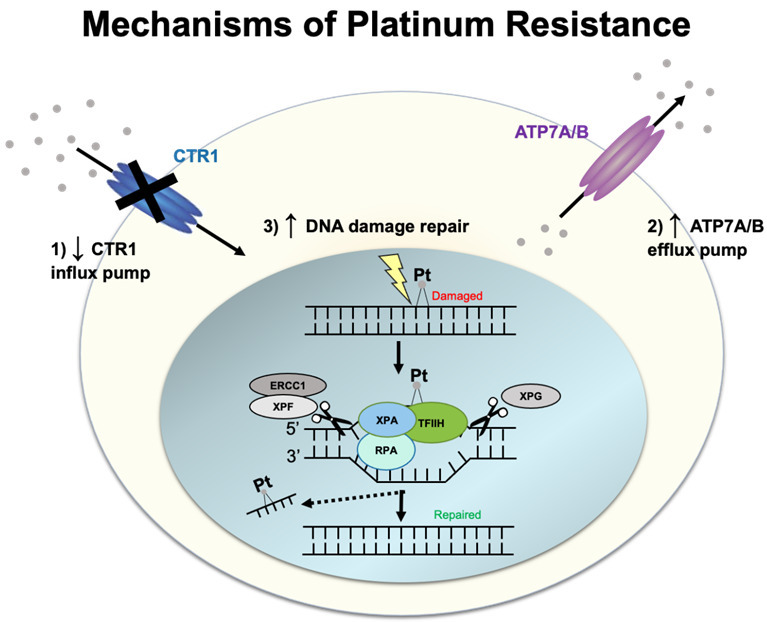
Schematic illustration of platinum resistance.

Cisplatin and carboplatin target cancer cells by forming adducts/crosslinks with DNA purine bases, with a preference for guanine. These crosslinks result in DNA damage that impedes proper genome replication, transcription, and triggers cell apoptosis^[9]^. A prevalent resistance mechanism centers around inhibiting the compound from reaching the DNA, which is mediated by efflux transporters. In addition, once DNA lesions are formed, DNA repair pathways are activated to fix DNA damage caused by the platinum agents, as described below [Figure 1].

### Influx and efflux transporters

One of the most widely accepted mechanisms of platinum resistance is the dysregulation of both influx and efflux pumps/transporters, which modulates the transport of platinum in ovarian cancer cells. When the first multidrug resistance transporter, an ATP-binding cassette transporter (also called ABCB1, P-glycoprotein, P-gp, or MDR1), was identified to have a function in the efflux of anticancer drugs out from cancer cells, it was also tested whether cisplatin is pumped out by P-gp^[10-13]^. However, it was identified that cisplatin is not a direct substrate of P-gp. Thus, it prompted the investigators to identify transporters that are similar to P-gp but have the ability to efflux cisplatin. This led to the discovery of the influx transporter, copper transporter 1 (CTR1), and efflux transporters, ATPase copper-transporting alpha and beta (ATP7A and ATP7B). Subsequent studies have shown that cisplatin-sensitive A2780 ovarian carcinoma cells have higher CTR1 expression than cisplatin-resistant A2780CP^[14]^ and that overexpression of CTR1 in A2780 cells increased the influx of cisplatin^[15]^. Clinically, patients who had cytoreductive surgery followed by platinum-based chemotherapy had CTR1 mRNA levels that correlated with platinum sensitivity^[16]^. Interestingly, cisplatin exposure in A2780 cells triggers rapid downregulation of CTR1, thereby inhibiting further cisplatin accumulation in the cells. This negative feedback loop has been proposed to result in resistance to cisplatin^[17]^. Cisplatin-induced downregulation of CTR1 is inhibited by the proteasome inhibitor bortezomib^[18]^. A clinical trial (NCT0107441) was recently completed to determine intraperitoneal bortezomib’s maximum-tolerated dose and dose-limiting toxicities when administered with carboplatin in an epithelial ovarian, fallopian tube, or primary peritoneal cancer. 

On the other hand, ATP7A/B efflux platinum^[19,20]^. However, only the silencing of ATP7B, and not in the silencing of ATP7A, in cisplatin-resistant cells (A2780-CP20 and RMG), resulted in increased sensitivity to cisplatin^[21]^. Clinical studies have also supported the prognostic value of ATP7B in ovarian cancer patients treated with cisplatin therapy^[22,23]^. In addition, a clinical study on 152 ovarian cancer patients has shown that genetic polymorphism in ATP7A is implicated in cisplatin resistance, and genetic polymorphisms in CTR1 is implicated in carboplatin resistance^[24]^.

### DNA repair

After entry of platinum drugs into the cytoplasm via CTR1, the platinum drugs enter the nucleus, where they form intrastrand and interstrand crosslinks with the DNA. Resistance to platinum occurs via DNA repair pathways. Single-strand DNA lesions are repaired by nucleotide excision repair (NER), and double-strand lesions are repaired either by homologous recombination (HR) or non-homologous end-joining (NHEJ) pathway^[25]^. In the NER pathway, the site of the DNA lesion is cleaved by ERCC1-XPF and XPG endonucleases to remove the DNA lesion. High ERCC1 expression is associated with platinum resistance in epithelial ovarian cancer but is not associated with patient survival^[26]^. Promoter methylation of mismatch repair (MMR) genes can also lead to cisplatin resistance by downregulating MMR-driven DNA damage response^[27]^. The MMR pathway carries DNA repair during DNA replication and recombination and specifically recognizes mismatched base pairing, insertions, and deletions^[28]^. In a study using cisplatin-resistant and MMR-deficient ovarian tumor xenografts caused by MLH1 promoter hypermethylation, treatment with the demethylation agent, 2’-deoxy-5-azacytidine, was shown to improve response to cisplatin and carboplatin^[27]^. 

### Emerging studies: other resistance mechanisms to platinum agents

More recently, other examples of platinum resistance have been explored. These include upregulating of de-ubiquitination of proteins targeted for proteasomal degradation^[29]^, increased cisplatin-induced autophagy^[30]^, and dependence on mitochondrial oxidative phosphorylation for energy supply^[31]^. Metabolic reprogramming and angiogenesis are hallmarks of cancer^[32]^ that provide tumor supply of nutrients and oxygen, energy efficiency, and drive cell survival; thus, they are also speculated to be involved in chemotherapy resistance. For instance, it has been recently shown that upregulation of the serine/threonine kinase Aurora-1 in cisplatin-resistant ovarian cancer increases glycolysis and suppresses cell senescence by stimulating the transcription factor sex determining region Y-box 8 (SOX-8)^[33]^. Another study has shown that fibrillin-1 (FBN1) is significantly upregulated in cisplatin-resistant ovarian cancer organoids and tissues and that FBN1 drives phosphorylation of VEGF2 and nuclear translocation of the transcription factor signal transducer and activator of transcription 2 (STAT2), which affects the expression of genes associated with STAT2-mediated glycolysis and angiogenesis^[34]^. A combination of FBN1 knockout and an antiangiogenic drug was demonstrated to improve cell sensitivity to cisplatin. Altogether, there are several emerging studies on mechanisms of platinum resistance in ovarian cancer. 

## MECHANISMS OF RESISTANCE TO PACLITAXEL

**Figure 2:**
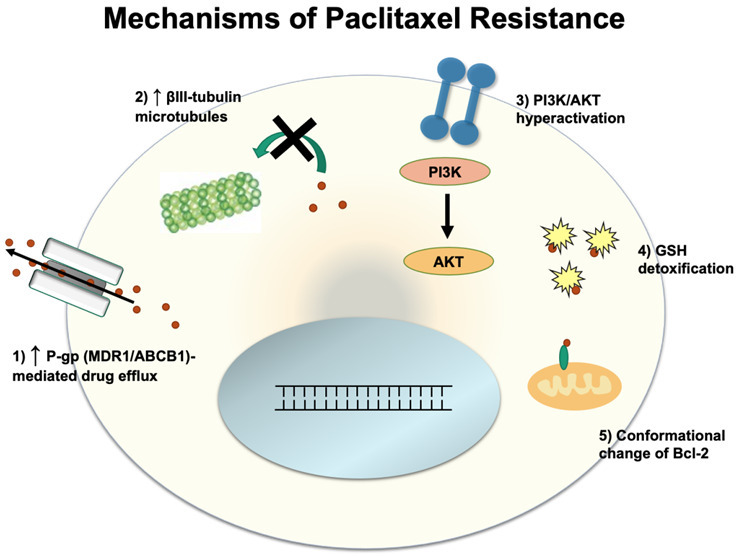
Schematic illustration of paclitaxel resistance.

Paclitaxel exerts its effect by binding to the β-subunit of tubulin and causing tubulin polymerization in the absence of GTP, a factor that is normally required for microtubule polymerization^[35]^. Once bound, paclitaxel stabilizes microtubules and prevents tubulin depolymerization. This inhibits the shortening of the microtubules during anaphase when it is necessary to pull apart sister chromatids, which results in cancer cell death^[36]^. Below, we discuss several mechanisms of resistance to paclitaxel [Figure 2].

### Efflux by P-glycoprotein

Like platinum-based chemotherapies, cancer cells can develop resistance to paclitaxel via efflux of paclitaxel out from the cancer cells. However, unlike cisplatin, paclitaxel is the major substrate of P-gp^[11,37]^. In order to prevent P-gp mediated efflux of paclitaxel, recent studies have shown that mutations in P-gp can suppress the efflux of paclitaxel^[38]^, and substitution of 14 conserved residues in homologous transmembrane helicases 6 and 12 with alanine resulted in reversal of P-gp from efflux to influx pump^[39]^, a potential future avenue for preventing efflux of paclitaxel. 

### Tubulin isotype composition

Paclitaxel is involved in the stabilization of microtubules, and as such, changes in microtubule composition can play a key role in determining cell susceptibility to paclitaxel. While tubulin has multiple isoforms, microtubules composed of mostly the βIII isoform show significantly lower stability compared to βI and βII microtubules^[40]^. Lower microtubule stability can counteract the microtubule-stabilizing effect of paclitaxel and thus allow cell division to occur despite paclitaxel activity. Paclitaxel-resistant ovarian tumors have been sampled to reveal up to a 4-fold increase in the proportion of βIII isoform, indicating that upregulation of βIII isoform production may confer paclitaxel resistance^[41]^.

### Phosphoinositide 3-kinase /protein kinase B pathway

The Phosphoinositide 3-kinase/protein kinase B (PI3K/AKT) pathway has been shown to be a key driver of metastasis and drug resistance in many different cancers, including ovarian cancer. Overactivation of this signaling pathway leads to the upregulation of factors involved in cell proliferation and migration. Mechanisms of PI3K/AKT hyperactivation include loss of function in PTEN (a negative regulator of PI3K/AKT), mutations in PI3K that confer constitutive activity, and hyperactivity of AKT^[40]^. These mutations cause the activation of pro-mitotic factors that overpower the anti-proliferative signals that result from paclitaxel dosing.

### Glutathione S-transferase 1

The detoxifying powers of the glutathione pathways are turned against paclitaxel in this resistance mechanism. In healthy cell conditions, Glutathione S-transferase P1 (GSTP1) production is inhibited by high PRMT6 activity, which inhibits the production of the GSTP1 precursor, G6PD. In this particular resistance mechanism, PRMT6 is downregulated in cancer cells, allowing for increases in G6PD production and higher levels of GSTP1 as a result. Thus, paclitaxel becomes sequestered by GSTP1 and detoxified in the cells before it can bind tubulin^[42]^. In the A2780 ovarian cancer cell line model, GSTP1 knockdown suppressed the invasion and migratory properties of the cells and sensitized the cells to cisplatin and carboplatin^[43]^. 

### B-cell lymphoma 2 family

In the B-cell lymphoma 2** (**Bcl-2) family, there are the pro-apoptotic factors, BAD and BAX, as well as the anti-apoptotic factors, Bcl-2, Bcl-XL, and Mcl-1^[44]^. Paclitaxel has been found to alter Bcl-2 activity, transforming it into a pro-apoptotic factor^[45]^. Mechanistically, paclitaxel mimics the nuclear orphan receptor Nurr77 to cause this effect. The Nurr77 and paclitaxel have structural similarities that explain this mimicry^[46]^. Just like Nurr77, paclitaxel binds to the N-terminal loop of Bcl-2 and induces a conformational change to expose the Bcl2-homology 3 domain. Upon phosphorylation of this conformational isoform, Bcl-2 changes from an anti-apoptotic factor to a pro-apoptotic factor by inducing cytochrome c release from the mitochondria^[47]^. Resistance to paclitaxel can be attributed to increases in anti-apoptotic Bcl-2 family members, as the anti-apoptotic factors inhibit FasL production, a ligand involved in cell death, by preventing its gene transcription^[44,48]^.

In addition, genetic differences in Bcl-2 have been attributed to varying rates of paclitaxel treatment success in patients. Recent studies have identified a T>C variant (RefSNP rs1801018) in the Bcl-2 sequence that is highly associated with paclitaxel resistance in multiple tumor types. In a retrospective genomic analysis of cancer patients, 73% of patients with T at location 21 did not respond to the platinum-paclitaxel combination therapy^[49]^. Although the exact mechanism facilitating this resistance is currently unknown, the data indicate a vital relationship between the Bcl-2 sequence and paclitaxel susceptibility and resistance.

### Paclitaxel and cisplatin cross-resistance

Cisplatin and paclitaxel are commonly used in conjunction since the two drugs bring cytotoxicity by distinct mechanisms and, thus, provide two unique barricades against uncontrolled cell growth^[9]^. Yet, as cells become resistant to cisplatin, they can also become resistant to paclitaxel. One such mechanism of this is through upregulation of cell survival pathways. Cells can combat cisplatin- and paclitaxel-induced apoptosis by upregulation of cell survival pathways, such as TNF/NFκB^[50]^. It is not yet known whether paclitaxel resistance mechanisms are able to confer cisplatin resistance, but the existence of common resistance mechanisms between the two drugs posits the idea.

## MECHANISMS OF RESISTANCE TO PARP INHIBITORS 

**Figure 3:**
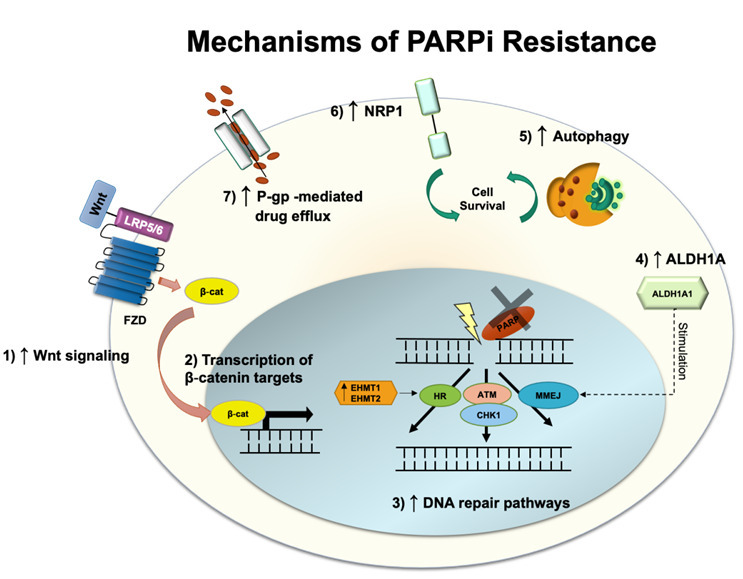
Schematic illustration of PARPi resistance. PARPi: Olymerase inhibitor.

Olaparib and niraparib are two PARPis recently approved by the FDA for targeted therapy in ovarian cancer in 2018 and 2020, respectively. These drugs are currently used as maintenance therapies or advanced treatments of ovarian cancer in platinum-sensitive patients who have undergone chemotherapy as first-line chemotherapy^[51]^. PARPi undermines single-strand DNA (ssDNA) damage repair, more specifically the base excision repair pathway, either by trapping PARP proteins on the DNA site of the lesion or by blocking PARP catalytic domain^[52]^. This prevents the binding of NAD+, a cofactor necessary for the post-translational modification, named PARPylation, onto targeted proteins to happen^[53]^. Unrepaired ssDNA breaks lead to double-strand DNA (dsDNA) breaks and genomic instability, triggering cell death^[54]^. Approximately 30 - 60% of the patients treated with PARPi respond to the treatment^[55]^; however, several cases of resistance have been reported. As the first FDA-approved PARPi, olaparib is the most investigated in terms of resistance mechanisms and will be further discussed below [Figure 3].

### DNA repair

Patients with impaired homologous recombination (HR) repair machinery often respond better to PARPi treatment. This combination leads to the accumulation of dsDNA breaks due to deficient DNA repair^[56]^. Hence, most resistance to olaparib and other PARPi has been linked to restoring DNA repair activity. Recently, it was shown that olaparib-resistant epithelial ovarian cancer cells harboring BRCA2 mutation had augmented microhomology-mediated end joining (MMEJ) pathway, which allows the cells to overcome PARP inhibition^[57]^. The increased expression of the epigenetic reader Bromodomain-containing protein 4 (BRD4) induces upregulation of aldehyde dehydrogenase 1 family member A1 (ALDH1A1), an enzyme associated with chemotherapy resistance through drug metabolism and inhibition of apoptosis signaling mediated by reactive oxygen species (ROS). ALDH1A1, in turn, is proposed to stimulate MMEJ and dsDNA repair. Thus, ALDH1A1 specific inhibition was shown to recover PARPi sensitivity. In another study, BRCA2-mutated high-grade serous ovarian carcinoma cells (HGSOC) resistant to olaparib exhibited histone-lysine-N-methyltransferase 1 and 2 (EHMT1/2) overexpression and consequent increase in histone H3 lysine 9 dimethylation, which was correlated with poorer overall survival^[58]^. As EHMT1/2 has been demonstrated to play a role in DNA repair by directly recruiting DNA damage response factors, the interruption of its activity leads to HR and NHEJ impairment, and disruption of the cell cycle, sensitizing these cells to PARPi. Nonetheless, no increase in cell death rates was observed when EHMT1/2 was inhibited. An alternative mechanism of resistance to olaparib in HGSOC cell lines independent of BRCA reverse mutation was demonstrated to be associated with increased activation of the Wnt signaling pathway^[59]^. Although Wnt drives different cell pathways by directly promoting β-catenin accumulation and upregulating β-catenin target-gene transcription, treatment with its inhibitor, pyrvinium pamoate, and olaparib abrogated DNA damage repair^[59]^. Furthermore, olaparib-resistant cell lines were shown to have enhanced HR and distal NHEJ induced either by Wnt-dependent or independent mechanisms.

### Cell cycle

The cell cycle regulation is critical for the proper execution of DNA repair^[60]^. Upon recognition of DNA damage, checkpoint kinases ATM and ATR phosphorylate downstream signaling pathways to determine whether to continue with cell cycle progression or pause for DNA repair^[61]^. HGSOC has universal p53 loss, which causes dysfunctional G1/S checkpoint, which makes the tumor be dependent on G2/M cell cycle arrest for DNA repair^[62]^. This G2/M cell cycle arrest is mediated by the cell cycle checkpoint kinase 1 (Chk1) and is activated by the DNA replication stress marker ATM and ATR, allowing HR to fix dsDNA breaks in the presence of collapsed replication forks. Moreover, Chk1 phosphorylates BRCA2 and RAD51 recombinase, assisting their nuclear translocation and interaction. RAD51, in turn, mediates invasion and pairing of the broken DNA strand with the homologous chromosome, used as the template for DNA recombination and repair^[63,64]^.

Inhibition of Chk1 with prexasertib has been demonstrated to attenuate olaparib-induced RAD51 translocation to the nucleus and foci formation leading to HR deficiency in BRCA wild-type or BRCA2-restored function HGSOC^[62]^. Thus, Chk1 inhibition was shown to enhance the sensitivity of ovarian cancer cells to DNA damage response inhibitors, such as PARPi, and the combination of both drugs decreases cell viability due to increased DNA damage and apoptosis. In addition, it promotes cell cycle progression to the M phase independent of the BRCA phenotype in cells once arrested in the G_2_/M checkpoint by olaparib treatment only. Importantly, over 90% of HGSOC have abnormal function of the tumor-suppressor TP53^[65]^, meaning that cells rely on the G_2_/M checkpoint in the absence of the G_1_/S. The combinatory treatment forces the cell to enter the M phase even in the presence of unrepaired DNA breaks, leading to cell death.

### Efflux transporters

Apart from DNA damage repair-associated mechanisms, other models were proposed to elucidate ovarian cancer resistance to olaparib. One such example is drug efflux pumps, and they have been widely explored to be involved in multi-drug resistance mechanisms. P-gp, which is encoded by* ABCB1* gene, is associated with first-line chemotherapy resistance to paclitaxel and other taxane drugs^[13]^. *ABCB1* gene expression and copy number were shown to be increased in paclitaxel-resistant ovarian cancer cells presenting cross-resistance to olaparib, and both drugs were actively effluxed from the cells^[66]^. Inhibition of P-gp with elacridar was able to resensitize the resistant cells to olaparib and paclitaxel, hence a combinatory treatment to improve sensitivity to PARPi. Similarly, targeting neuropilin-1 (NRP1), a transmembrane receptor that contributes to cell contact evasion and tumorigenesis in ovarian tumors, with the miRNA miR-200c, induces sensitization of resistant SKOV3 ovarian cancer cell lines (BRCA wild-type) to olaparib^[56]^. In these cells, NRP1 was demonstrated to be present in higher levels compared to the sensitive UWB1.289 BRCA1-null cell line or the partially resistant UWB1.289 in which BRCA1 function was restored.

### Autophagy

Regardless of the BRCA phenotype, clinical trials (NCT01847274; NCT02354586) have demonstrated that niraparib leads to better outcomes in patients with wild-type HR^[67]^. Although niraparib resistance in ovarian tumors has been reported, whether similar pathways drive this as for other PARPi remains to be elucidated. As the FDA approval of niraparib happened more recently, only a few studies have explored its specific resistance mechanisms to date. Nonetheless, a recent study treating a variety of ovarian cancer cell lines, including the BRCA wild-type OVCAR8 and HEY cells, with four PARPis, olaparib, niraparib, rucaparib, and talazoparib, demonstrated increased autophagy activation^[68]^. Autophagy has been described as a common resistance mechanism to overcome anticancer drugs by providing the energy supplies necessary for cell survival under stressful conditions. It also contributes to the hypoxic microenvironment and metabolic stress, two components modulated by cancer cells to escape cell death. Hence, targeting autophagy for downregulation may improve patient response to PARPi. Combining olaparib and inhibitor that can target autophagy may increase ATP phosphorylation and ROS formation. As a consequence, increased cell death and proliferation suppression were observed after the combinatory treatment. Further studies are needed to elucidate whether autophagy itself is enough to drive cell survival in cells containing high genomic instability.

In summary, ovarian cancers have several resistance mechanisms to PARPis. These include stimulating DNA damage response, preventing genomic instability either by upregulating Wnt signaling, inducing EHMT1/2 overexpression, or increasing ALDH1A1 levels. Alternatively, olaparib might be actively effluxed from the cells using the P-gp pump. Resistance to olaparib and niraparib has also been attributed to PARPi-mediated-autophagy, which contributes to the metabolic modulation of cancer cells and prolonged cell survival. 

## MECHANISMS OF RESISTANCE TO BEVACIZUMAB

**Figure 4:**
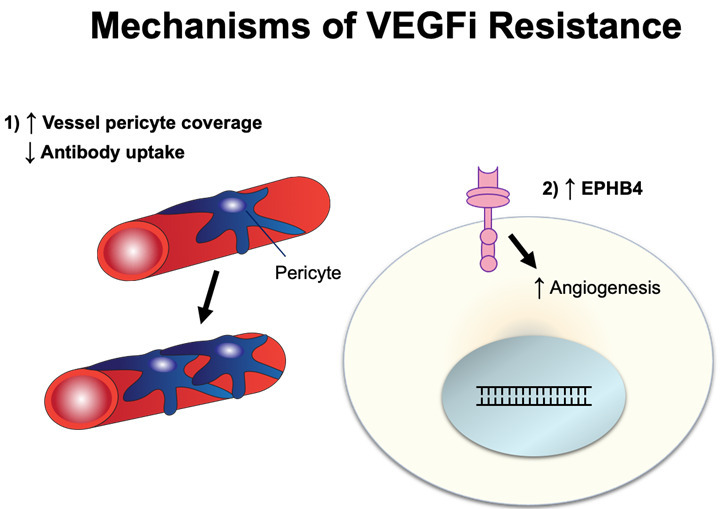
Schematic illustration of VEGFi resistance. VEGFi: Vascular endothelial growth factor inhibitor.

Bevacizumab is a humanized monoclonal IgG1 antibody designed to target VEGF^[69]^ and was recently approved as maintenance therapy for ovarian cancer in 2020. VEGF, an angiogenic growth factor, is often produced by cancer cells to drive blood vessel growth and, consequently, divert nutrients directly to tumors^[70,71]^. Bevacizumab binds directly to circulating VEGF, preventing it from interacting with VEGF-receptors, thus inhibiting angiogenesis by starving the tumor from nutrients. Resistance mechanisms against bevacizumab have been discussed in other cancer settings^[72,73]^. Here, we discuss resistance mechanisms to bevacizumab in ovarian cancer [Figure 4].

### Bevacizumab-induced decreased antibody uptake, increased vessel pericyte coverage, and angiogenesis

When angiogenesis occurs in solid tumors, it forms a defective vasculature with increased tumor permeability. This alters the tumor microenvironment and affects intra-tumoral drug delivery. When ovarian cancer SKOV3 mouse xenograft was evaluated for antibody uptake using PET imaging of ^89^Zr-bevacizumab, it was observed that bevacizumab treatment decreased tumor uptake and lessened intra-tumoral accumulation of bevacizumab with increased vessel pericyte coverage^[74]^. Pericyte promotes endothelial cell survival via activation of VEGF-A, and, therefore, may contribute to the resistance to VEGFi^[75,76]^. In addition to increased vessel pericyte coverage, ovarian cancer cells can circumvent bevacizumab via angiogenesis. For instance, the crosstalk between endothelial cells and ovarian cancer cells can activate the PI3K/Akt pathway and stimulate the proangiogenic factor FGF2, overcoming VEGF-dependent vascularization as an evasive mechanism^[77]^.

### Ephrin type-B receptor 4

Ephrin type-B receptor 4 (EphB4) is a tyrosine kinase receptor with a functional role in blood vascular morphogenesis and angiogenesis^[78]^. While the exact resistance mechanism has not yet been determined, EphB4 is overexpressed in bevacizumab-resistant ovarian cancer SKOV3 xenograft and co-administration of bevacizumab with the EphB4 blocker, NVP-BHG712, results in reversal of resistance and inhibition of tumor growth^[79]^.

The mechanisms by which cancer cells develop resistance to bevacizumab have yet to be fully characterized. While the resistance mechanisms mentioned above have been identified, the future discovery of additional resistance mechanisms should be anticipated in the ovarian cancer setting.

## CONCLUSION

In this review, we have described mechanisms of drug resistance against platinum, paclitaxel, olaparib, niraparib, and bevacizumab in the context of ovarian cancers. While we know many resistance mechanisms against platinum and paclitaxel, we continue to discover novel resistance mechanisms due to technological advancement in the field. There is currently a major interest in the field to understand the resistance mechanisms of new ovarian cancer treatments such as olaparib, niraparib, and bevacizumab. We anticipate more new insights and discoveries in novel resistance mechanisms as well as novel approaches to covercome drug resistance (i.e., nanomedicine^[80]^) in the next few years. 

## DECLARATIONS

### Copyright

© The Author(s) 2022.
